# Cerebral Dopamine Neurotrophic Factor (CDNF) Acts as a Trophic Factor Promoting Neuritogenesis in the Dorsal Root Ganglia (DRG) Neurons Through Activation of the PI3K Signaling Pathway

**DOI:** 10.1111/jnc.70194

**Published:** 2025-08-19

**Authors:** Raphael de Siqueira Santos, Flávia Natale Borba, Dahienne Ferreira de Oliveira, Marcelo Felippe Santiago, Alexandre Martins do Nascimento, Deborah Schechtman, Debora Foguel

**Affiliations:** ^1^ Instituto de Bioquímica Médica Leopoldo De Meis Universidade Federal Do Rio de Janeiro Rio de Janeiro Brazil; ^2^ Instituto de Biofísica Carlos Chagas Filho Universidade Federal do Rio de Janeiro Rio de Janeiro Brazil; ^3^ Departamento de Bioquímica, Instituto de Química Universidade de São Paulo São Paulo Brazil

**Keywords:** cerebral dopamine neurotrophic factor, dorsal root ganglia, KDEL‐receptor, nerve growth factor, PI3K/AKT

## Abstract

The cerebral dopamine neurotrophic factor (CDNF) is a neurotrophic factor extensively studied in the central nervous system because of its neuroprotective effects; however, its role in the peripheral nervous system (PNS) remains less explored. In this study, we used primary dorsal root ganglia (DRG) explants to investigate the neuritogenic potential of exogenous CDNF, as well as its neuroprotective activity under trophic factor deprivation. Our findings demonstrate that CDNF‐mediated neuroprotection remains unaffected by the addition of a Trk (tropomyosin receptor kinase) inhibitor or anti‐nerve growth factor (NGF) antibody, indicating that CDNF's neurotrophic activity is independent of TrkA signaling. Furthermore, CDNF binding to KDEL‐receptor (KDEL‐R) was essential for its protective effect, as the CDNF variant lacking the KDEL‐R binding sequence (CDNF‐ΔKTEL) displayed no significant neuroprotection. Additionally, the simultaneous administration of NGF and CDNF to DRG explants resulted in an additive enhancement of their trophic activities. Notably, both CDNF‐ and NGF‐induced neurotrophic effects were PI3K‐dependent, reinforcing the role of this signaling pathway in their mechanisms of action. Taken together, our findings highlight CDNF's crucial role in the PNS, ensuring that NGF‐independent neurogenesis can occur. This suggests that CDNF could be further explored in conditions where NGF levels are low or where NGF signaling inhibition is desirable, such as in chronic pain management.

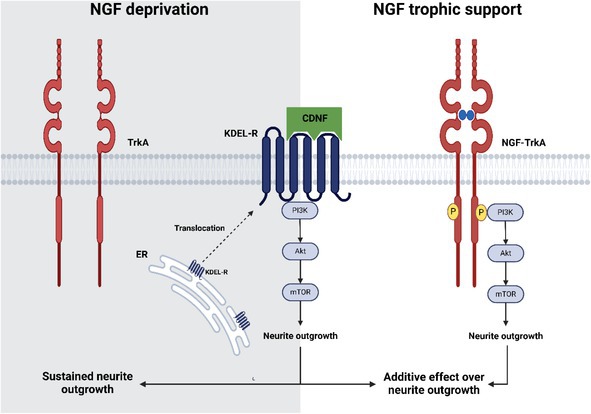

## Graphical abstract

Cerebral dopamine neurotrophic fator (CDNF) comprises a novel family of neurotrophic factors. CDNF is an extensively studied neurotrophic factor in the central nervous system (CNS); however, its role in the peripheral nervous system (PNS) remains less explored. We show for the first time that CDNF is capable of promoting neuroprotection, viability and neuritogenesis in PNS cells, and that its neuroprotective activity persists under stress conditions. The neurotrophic activity of CDNF is independent of signaling from the classical nerve growth factor (NGF) receptor, TrkA. However, binding of CDNF to the KDEL‐1 receptor (KDEL‐R) was essential for its protective effect, since the CDNF variant with a deletion of the last four amino acids (KDEL‐R‐binding region) called CDNF‐ΔKTEL failed to exert neurotrophic actions. Under NGF deprivation, KDEL‐R translocates from the endoplasmic reticulum to membrane plasmatic to bind to CDNF and, consequently, supporting CDNF effects. Simultaneous administration of NGF and CDNF promoted an additive effect of their trophic activities. These effects were PI3K‐dependent, reinforcing the role of this signaling pathway in its mechanisms of action. In summary, this work pioneeringly demonstrates a crucial role for CDNF in the PNS, becaming CDNF a possible replacement for NGF and opening a window for understanding the neurochemical mechanisms of PNS development and expanding its therapeutic applications in neurodegenerative conditions.

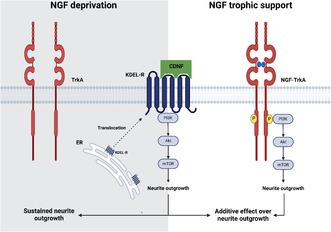

**Abbreviations**
AKT: protein kinase B
BAX: BCL2‐associated X protein
Bcl‐2: B‐cell lymphoma protein 2
BDNF: brain‐derived neurotrophic factor
CDNF: cerebral dopamine neurotrophic factor
Chop: CREBP‐homologous protein
CNS: central nervous system
COPI: coat protein I
DAPI: 4′,6‐diamidino‐2‐phenylindole dihydrochloride
DRG: dorsal root ganglia
ER: endoplasmic reticulum
ERK: extracellular signal‐regulated kinase
FITIC: fluorescein isothiocyanate isomer I
GPCRs: G‐protein‐coupled receptors
GRP‐78: 78 kDa glucose‐regulated protein
I/R: ischemia–reperfusion
KDEL: lysine, aspartic acid, glutamic acid, and leucine
KDEL‐R: KDEL receptor
KTEL: lysine, threonine, glutamic acid, and leucine
MANF: mesencephalic astrocyte‐derived neurotrophic factor
mTOR: mammalian target of rapamycin
NFs: neurotrophic factors
NGF: nerve growth factor
NMR: nuclear magnetic resonance
PD: Parkinson's disease
PI3K: phosphatidylinositol‐3‐kinase
PKA: cyclic AMP‐dependent protein kinase
PLCγ: phospholipase Cγ
PNS: peripheral nervous system
RRID: research resource identifier
RTDL: arginine, threonine, aspartic acid, leucine
SCG: superior cervical ganglion
TrkA: tropomyosin receptor kinase A
Trki: tropomyosin receptor kinase inhibitor
UPR: unfolded protein response
WGA: wheat germ agglutinin

## 1 Introduction

Neurotrophic factors (NFs) are molecules that promote neurogenesis by regulating neuronal proliferation, growth, survival, migration, maturation, and differentiation in both the central and peripheral nervous systems (CNS and PNS). Additionally, they play a key role in modulating synaptic plasticity (Lane et al. 2008; Skaper 2018; Huttunen and Saarma 2019). NFs carry out these functions during early neural development and throughout adulthood, while also contributing to nerve regeneration. This broad spectrum of biological activities highlights their significant therapeutic potential (Bartkowska et al. 2010; Ledda and Paratcha 2016; Bothwell 2019; Gu et al. 2025; Yang et al. 2025; Numakawa and Kajihara 2025).

Cerebral dopamine NF (CDNF) belongs to a recently characterized unconventional family NFs, which also includes mesencephalic astrocyte‐derived NF (MANF) (Lindholm et al. 2007; Bothwell 2014; Ibáñez and Andressoo 2017). These proteins primarily reside in the endoplasmic reticulum (ER) but can also be secreted, where they play key roles in paracrine and autocrine signaling (Petrova et al. 2003; Albert and Airavaara 2019; Danilova et al. 2019).

Numerous studies have explored the mechanisms of action of MANF and CDNF in the CNS (Petrova et al. 2003; Lindholm et al. 2007; Voutilainen et al. 2009; Huttunen and Saarma 2019; Albert et al. 2021; Er and Airavaara 2023). CDNF has been shown to effectively mediate neuroprotection in models of Parkinson's disease (PD), Huntington's disease, stroke, amyotrophic lateral sclerosis, and cell death associated with ER stress (Eesmaa et al. 2022; Caglayan et al. 2023; De Lorenzo et al. 2023; Stepanova et al. 2023; Grote et al. 2024).

Previously, our group determined the three‐dimensional structure of CDNF using NMR (Latge et al. 2015). The protein consists of two well‐defined domains composed of α‐helices, stabilized by four disulfide bonds. At the C‐terminal end, CDNF contains the KTEL sequence (lysine, threonine, glutamic acid, and leucine), which closely resembles the KDEL motif (lysine, aspartic acid, glutamic acid, and leucine) found in proteins that bind to the KDEL receptor (KDEL‐R), such as Grp78, calnexin, and MANF, among others.

KDEL‐R is a seven‐transmembrane protein primarily localized to the Golgi under steady‐state conditions. Its main function is to retrieve proteins from the Golgi apparatus back to the ER through a coat protein I (COPI)‐dependent pathway, preventing their exocytosis (Capitani and Sallese 2009; Giannotta et al. 2015). Although some studies have reported the constitutive presence of KDEL‐R at the cell membrane (Henderson et al. 2013; Lindahl et al. 2014; Becker et al. 2016), certain conditions, such as ER stress, can trigger its migration from the ER to the plasma membrane. Once at the cell surface, KDEL‐R binds to extracellular ligands containing KDEL or KDEL‐like sequences, initiating a cellular response (Yoshida 2007; Jia et al. 2021, 2024).

Several studies have demonstrated that MANF and CDNF, as ER‐resident proteins, function as modulators of the unfolded protein response (UPR), a cellular mechanism activated under ER stress conditions such as calcium depletion, protein misfolding, and energy or glucose deprivation, to restore homeostasis and prevent early apoptosis (reviewed in Lõhelaid et al. 2024). Although the precise mechanism by which MANF and CDNF regulate the UPR remains unclear, multiple studies have investigated this critical question. MANF and CDNF interact with GRP78, a central regulator that controls the three branches of the UPR (Yan et al. 2019; Graewert et al. 2024). Additionally, CDNF and MANF modulate the mRNA levels of several UPR‐associated genes in different cellular models, including pro‐apoptotic proteins such as CHOP (C/EBP‐homologous protein), Bcl‐2/Bax, and caspase‐3 (Eesmaa et al. 2021, 2022; Arancibia et al. 2018).

Interestingly, our group reported the effects of CDNF in non‐neural cells, demonstrating that exogenous CDNF can promote cardiomyocyte survival by protecting against ER stress and ischemia–reperfusion (I/R) injury. This protective effect occurs via activation of the phosphatidylinositol‐3‐kinase (PI3K) and protein kinase B (AKT) signaling pathway (Maciel et al. 2021). Moreover, a CDNF construct lacking the KTEL sequence (CDNF‐ΔKTEL) failed to exhibit cardioprotective activity, suggesting that the KDEL‐R translocated to the plasma membrane plays a crucial role in this process (Maciel et al. 2021).

To date, only a limited number of studies have investigated the trophic effects of CDNF on PNS neurons (Cheng et al. 2013; Liu et al. 2014). Notably, in an in vivo rat model of sciatic nerve transection, collagen nanotubes containing mesenchymal stem cells transduced with CDNF successfully restored sciatic nerve cells, leading to functional recovery (Liu et al. 2014). Additionally, *Cdnf*−/− mice exhibited age‐related degeneration of enteric neurons in the submucosal plexus, resulting in impaired gastrointestinal motility—a prodromal symptom of PD (Lindahl et al. 2020). However, the mechanisms underlying this process remain poorly understood.

In this study, we investigate the neurotrophic properties of exogenously added CDNF and its variant, CDNF‐ΔKTEL, in dorsal root ganglia (DRG) explants and dissociated cell cultures, comparing their effects to those of nerve growth factor (NGF). NGF is a well‐characterized neurotrophin known for its role in promoting the survival and neurite outgrowth of DRG neurons (Patel et al. 2003). NGF exerts its effects by binding to its high‐affinity receptor, tropomyosin receptor kinase A (TrkA), a tyrosine kinase that undergoes autophosphorylation upon NGF dimer binding. This activation triggers several downstream signaling pathways, including PLCγ, ERK, and PI3K (Huang and Reichardt 2003; Zhang et al. 2017), with PI3K playing a pivotal role in mediating NGF's neurotrophic effects (Song and Yoo 2011; Wong et al. 2015; Sang et al. 2018).

Our findings demonstrate that CDNF supports neuritogenesis and provides neuroprotection to DRG neurons, like NGF, whereas CDNF‐ΔKTEL is inactive. This suggests that the KDEL‐R at the plasma membrane plays a crucial role in mediating CDNF's effects. Supporting this hypothesis, we observed that KDEL‐R translocates to the plasma membrane of trophically deprived DRG dissociated cells and colocalizes with fluorescently labeled CDNF (^FITC^CDNF), further reinforcing KDEL‐R as a putative receptor for CDNF in PNS neurons. Additionally, we identified the PI3K/AKT/mTOR pathway as a key mediator of the trophic effects of both CDNF and NGF. Notably, the simultaneous application of NGF and CDNF to DRG explants resulted in an additive enhancement of their trophic activities. Together, these findings reveal novel neurotrophic properties of CDNF in the PNS, paving the way for new therapeutic strategies targeting nerve injuries.

## 2 Materials and Methods

### 2.1 CDNF and CDNF‐ΔKTEL Expression and Purification

CDNF and CDNF‐ΔKTEL (CDNF without the last four residues KTEL) were expressed and purified as previously published (Latge et al. 2015; Maciel et al. 2021). Briefly, proteins were eluted from a 5 mL Hitrap SP XL column (GE Healthcare, Chicago, IL, USA) equilibrated with 25 mM MES at pH 6.0. The retained material was eluted with a linear gradient of 0–1 M NaCl and 25 mM MES at pH 6.0. Fractions containing the protein, as checked by absorption at 280 nm, were submitted to a further purification step in gel filtration molecular exclusion chromatography, Sephacryl S‐100 column (16 × 100 Mm—GE Healthcare), equilibrated with 25 mM MES, 150 mM NaCl at pH 6.0. A prominent, defined peak around the expected elution volume was observed according to the calculated molecular mass of CDNF and CDNF‐ΔKTEL (~18 kDa). Protein purity was evaluated by running samples in denaturing polyacrylamide gel electrophoresis. After purification, proteins were subjected to a sample endotoxin elimination step, necessary for their use in cell cultures, using Pierce high‐capacity endotoxin removal resin (Cat. No. 88274 Thermo Fisher Scientific). According to the Manufacturer's protocol.

### 2.2 Animals and Bioethics

Neonatal C57BL/6 mice (IMSR_JAX:000664) with an age ranging from postnatal days 0 to 3, from both sexes, were used in in vitro experiments. The required sample size for each experiment was determined by using the online tool “powerandsamplesize.com” assuming a desired statistical power of 80%, a medium effect size (0.5), and a 95% confidence interval. To estimate the effect size, we used neurite length data obtained under NGF trophic support conditions (mean = 803.60 μm; SEM = 79.04 μm). On the basis of these parameters, we determined that three independent experiments would be sufficient to allow for multiple comparison analyses. Animals come from the Transgenic Animal Laboratory‐LAT (Federal University of Rio de Janeiro). All animals were kept in 12 h light/dark cycles with free access to food and tap water in an isolated animal room. The experimental protocol involving neonatal mice and adult mice used for mating was performed following the National Institutes of Health Guidelines for the Care and Use of Laboratory Animals and approved by the Ethics Committee for the Use of Animals in Research from the Federal University of Rio de Janeiro (CEUA—UFRJ/Health Sciences Center 135/21).

### 2.3 DRG Explants and Dissociated Cell Cultures

DRG explants were obtained by rapid decapitation of neonatal mice, followed by dissection through the ventral incision as previously published (de Siqueira‐Santos et al. 2019). Up to 10 different births were used in the in vitro studies in each section. About six neonatal animals (P0–P3) were used in each n, with each group being reproduced in at least triplicate cultures from independent experimental plates. Thus, we performed a triplicate (three wells) in each plate (one ganglion per well), totaling 60 neonates used. The culture plates of different groups were marked only by the date of the experiment, and the person responsible for acquiring the images for analysis or quantifying the data did not have access to these codes before. Explants were placed on laminin (10 μg/mL, cat. no. 23017‐015 Life Technologies) coated coverslips in DMEM/F12 culture medium (cat. no. 11330‐032 Gibco) supplemented with NGF 30 ng/mL (or stated in Figure legends, cat. no. 13257‐019 Life Technologies) (adapted from de Siqueira‐Santos et al. 2019, 2023), CDNF or CDNF‐ΔKTEL 1.8 μg/mL or 18 μg/mL (or stated in Figure legends) for 72 h at 37°C and 5% CO_2_. Dissociation of DRG was performed by trypsinization with 0.05% trypsin (cat. no. 25300062 Gibco) for 12 min at 37°C. Cell suspensions were cultured in the same conditions as explants. In the experiments shown in Figure 4D–G (dose–response with LY‐294002, a PI3K inhibitor) and Figure S2 (concentration curve with the pan‐Trk inhibitor GNF‐5837), a different recombinant NGF (cat. no. 450‐01, PeproTech) was used. This NGF shares approximately 85% sequence identity with the recombinant NGF used in the other experimental conditions.

### 2.4 Deprivation Experiments and Signaling Pathways

For NGF deprivation experiments, DRGs were kept for 24 h in the presence of NGF. Culture media were then replaced by medium only (deprived group), or media containing NGF (30 ng/mL or as stated in Figure legends), CDNF, or CDNF‐ΔKTEL (both at 1.8 μg/mL or 18 μg/mL or stated in Figure legends) for an additional 48 h.

To unravel the signaling pathways involved in neuritogenesis, we incubated DRG cultures with wortmannin (400 nM; Cat. No. W3144 Merck) or LY294002 (From 4 to 400 nM); cat. No. S1105 Selleckchem, both are PI3K pathway inhibitors; rapamycin (20 nM; Cat. No. S‐015 Merck), a mTOR pathway inhibitor; GNF‐5837 (20 μM; Cat. No. S7519 Selleckchem), a pan‐Trk inhibitor; and anti‐NGF antibody (10 μM; Cat. No. AF256‐NA RD Systems, RRID:AB_2106738) to inhibit NGF binding to its receptor.

### 2.5 Quantification of Neuritogenesis

Morphometric analyses were based on previous studies (de Siqueira‐Santos et al. 2019, 2023). Neurite outgrowth was evaluated by imaging eight random regions or by measuring the average neurite length (the distance from the beginning to the end of each neurite) to obtain an average value per DRG explant. To quantify the neuritic area, the area occupied by the central ganglia was subtracted from the total area occupied by ganglia plus neurites. Images were acquired using the EVOS M5000 microscope, and analyses were carried out by Image‐Pro Plus software (V6.0.0.260). Neurite complexity analysis was performed by counting the intersections between the neurites and concentric circles originated by analyzing the Sholl plugin of the Image‐J 1.48v program (US National Institute of Health). To better identify statistical differences, after the Sholl analysis, the areas under the curve (AUC) were calculated.

### 2.6 LDH Assay

Lactate dehydrogenase activity (LDH) was measured in the culture supernatant by CytoTox96 Kit (cat. no. G1780 Promega, RRID:AB_2687865) according to the manufacturer's instructions. Briefly, 50 μL of culture supernatant was incubated with 50 μL of substrate mix in a 96‐well plate at room temperature and protected from light. After 30 min, 50 μL of stop solution was added, and the plate was read at 490 nm on a SpectraMax Paradigm reader.

### 2.7 Immunocytochemistry

At the end of incubation times, DRG cultures were carefully washed with PBS and fixed with 4% paraformaldehyde for 15 min. Prior to immunolabeling, coverslips were washed three times for 5 min each with PBS, permeabilized with 0.3% Triton‐X‐100 (cat. no. T9284 Sigma‐Aldrich) for 10 min at room temperature, blocked with 5% bovine serum albumin (cat. no. A3294 Sigma‐Aldrich) in PBS for 1 h, and then incubated overnight at 4°C with primary antibodies such asTuj‐1 (1:400 cat. no. MA1‐118 Life Technologies, RRID:AB_2536829), S‐100β (1:100 cat. no. s2532 Sigma‐Aldrich, RRID:AB_477499), NeuN (1:100 cat. no. MAB377 Millipore, RRID:AB_2298772), and KDEL‐R1 (1:500 cat. no. SAB5200004 Sigma‐Aldrich, RRID:AB_3697651). Cells were then incubated with secondary Alexa Fluor antibodies 546, 488, or 680 (1:800, cat. no. A‐11030, RRID:AB_2737024; cat. no. A‐11029, RRID:AB_2534088; cat. no. A10038, RRID:AB_11180593, respectively, Life Technologies) at room temperature for 90 min. Nuclei were stained with 4′,6‐diamidino‐2‐phenylindole dihydrochloride for 10 min (DAPI cat. no. D1306 Thermo Fisher Scientific, RRID:AB_2629482), followed by two washes with PBS. Samples were mounted on microscope slides with n‐propyl gallate (cat. no. P3130 Sigma‐Aldrich) in 80% glycerol (cat. no. G9012 Sigma‐Aldrich). To determine dead cells in the dissociated culture, In Situ Cell Death Detection Kit, TMR Red was added along with the secondary antibody (cat. no. 12156792910 MERCK, AB_2315392). DRG explants were visualized and photographed under fluorescence light microscopy using Evos M5000 or confocal Elyra SR‐SIM (Zeiss) microscope.

### 2.8 Assessment of CDNF‐KDEL‐R Binding

Dissociated DRG neurons were maintained in culture for 24 h in a medium containing NGF (30 ng/mL), following NGF deprivation for an additional 24 h to stress the cells and to induce KDEL‐R migration to the plasma membrane (Martire et al. 1996; Raykhel et al. 2007; Trychta et al. 2018; Maciel et al. 2021). To evaluate the interaction of the KDEL‐R with CDNF or CDNF‐ΔKTEL, 1 μmol/mL of each protein was labeled with Fluorescein isothiocyanate isomer I (FITC) (cat. no. F4274 Sigma‐Aldrich) and incubated in the last 15 min of the deprivation period. Before fixation, cells were incubated with wheat germ agglutinin (WGA cat. no. ICN790162 Thermo Fisher Scientific, RRID:AB_2334867), a cell membrane marker. Cells were then fixed with 2% paraformaldehyde for 10 min at room temperature under constant agitation, washed with PBS, and incubated with a blocking solution (5% BSA, in PBS pH 7.4) for 1.5 h. Afterward, primary antibody against KDEL‐R (anti‐KDEL‐R1,1:500; Monoclonal Anti‐KDEL‐R1 antibody produced in mouse, cat SAB5200004, Sigma‐Aldrich), secondary Alexa Fluor 680 (1:500, mouse) and 4′,6′‐diamino‐2‐phenylindole (DAPI, 1:5000) were added and incubated for an additional 15 min. Next, coverslips were mounted on a glass slide in the mounting medium (ProLong Gold Antifade Mountant cat. no. P36934 Thermo Fisher Scientific, SCR_015961). Images were acquired in an Elyra confocal microscope equipped with an × 100 oil immersion objective.

### 2.9 Image Processing and Statistical Analysis

Images were processed using Image‐J 1.48v software (US National Institute of Health), and all data were submitted to statistical analysis using GraphPad Prism (10.3.0). Raw data were assessed for normality using the Kolmogorov‐Smirnov test and fitting to the normal distribution curve. Parametric data were analyzed using One‐Way ANOVA, with Tukey's post hoc test for differences between groups, whereas non‐parametric data were analyzed using Kruskal–Wallis, with Dunn's post hoc test. Data in graphs are presented as mean ± standard error of the mean (SEM). *p*‐values < 0.05 were considered statistically significant. No test for outliers was conducted. Each analysis consisted of a minimum of three biological replicates, each defined by at least three technical replicates. A detailed description of the statistical approach and the *p*‐values for each comparison are presented in Data S1.

## 3 Results

### 3.1 CDNF Exhibits Neurotrophic Activity by Promoting Neurite Outgrowth in DRG Explants

We first investigated the neurotrophic activity of CDNF in sustaining DRG explants in culture, comparing its effects to those of NGF. To evaluate the neurotrophic properties of these factors, we performed morphometric analysis and cell viability assays on DRG explants derived from P0–P3 neonatal mice treated with either NGF or CDNF.

**FIGURE 1:**
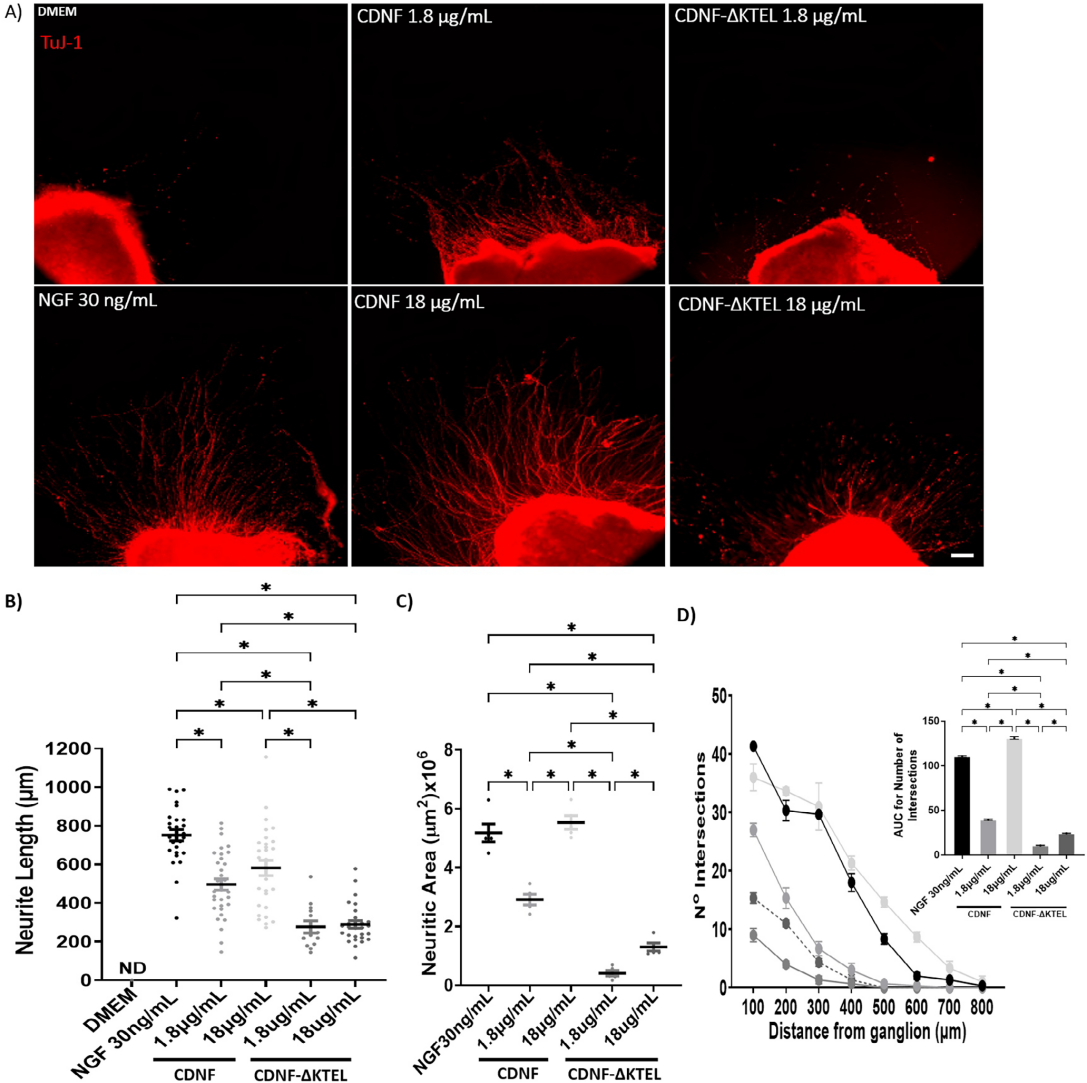
CDNF, like NGF, promotes neurite outgrowth in cultured dorsal root ganglia explants, whereas CDNF‐ΔKTEL does not exhibit this effect. (A) Representative images of dorsal root ganglia for 3 days in DMEM/F‐12 medium supplemented or not with nerve growth factor (NGF, 30 ng/mL), cerebral dopamine neurotrophic factor (CDNF, 1.8 or 18 μg/mL), or CDNF‐∆KTEL (1.8 or 18 μg/mL). The cytoskeleton of neurons was labeled with anti‐TuJ‐1 antibody in red. (B–D) Quantification of neurite length, neuritic area, and number of intersections (neurite complexity) from the ganglion body, respectively. Area under the curve (AUC) was shown in the inset (panel D). Data are presented as mean ± SEM from *n* = 6 independent experiments. One‐way ANOVA, with Tukey's post hoc test for significance. *p < 0.05 indicates statistical significance. Scale bar = 100 μm.

Neuritogenesis was significantly impaired in DRG explants cultured in DMEM/F12 without trophic factors or in the presence of CDNF‐ΔKTEL, a version of CDNF without the last four residues KTEL that interact with KDEL‐R (Figure 1). In contrast, NGF (30 ng/mL) induced robust neurite outgrowth, a response similarly observed with CDNF at both 1.8 μg/mL and 18 μg/mL concentrations (Figure 1A). Cultures supplemented with 30 ng/mL NGF exhibited an average neurite length of 750 μm, whereas 1.8 μg/mL and 18 μg/mL CDNF resulted in average lengths of 500 μm and 600 μm, respectively. In contrast, CDNF‐ΔKTEL supported only 200 μm of neurite growth (Figure 1B). As shown in Figure S1, CDNF concentrations either lower or higher than these two reference values were found to be, respectively, ineffective or even toxic to the DRG explants. Therefore, most subsequent experiments were conducted using 18 μg/mL.

Regarding neurite coverage, both 18 μg/mL CDNF and 30 ng/mL NGF induced a maximum occupied area of about 5.2 × 10^6^ μm^2^, whereas CDNF‐ΔKTEL (1.8 and 18 μg/mL) resulted in a significantly reduced area of just 2.9 × 10^6^ and 1.3 × 10^6^ μm^2^ on average, respectively (Figure 1C). Similarly, analysis of neurite network complexity, measured by the number of intersections, showed that 18 μg/mL CDNF (light gray symbols) and 30 ng/mL NGF (black symbols) promoted comparable levels of complexity. Conversely, treatment with 1.8 μg/mL CDNF (medium gray symbols) or CDNF‐ΔKTEL (darker gray symbols) resulted in fewer neurite intersections (Figure 1D). These data are better visualized when expressed by the area under the curve (AUC; inset).

Taken together, these results suggest that similar to NGF, CDNF exerts potent neurotrophic effects on DRG neurons of the PNS, albeit at higher concentrations. This activity appears to be mostly mediated by the KDEL receptor (KDEL‐R), as the neuritogenic effect was markedly diminished in the CDNF variant lacking the C‐terminal KTEL motif.

### 3.2 CDNF and NGF Additively Enhance Neurite Outgrowth in DRG Explants

Having established that CDNF promotes neurite outgrowth comparable to NGF, we next investigated whether combining both factors would enhance this effect.

**FIGURE 2:**
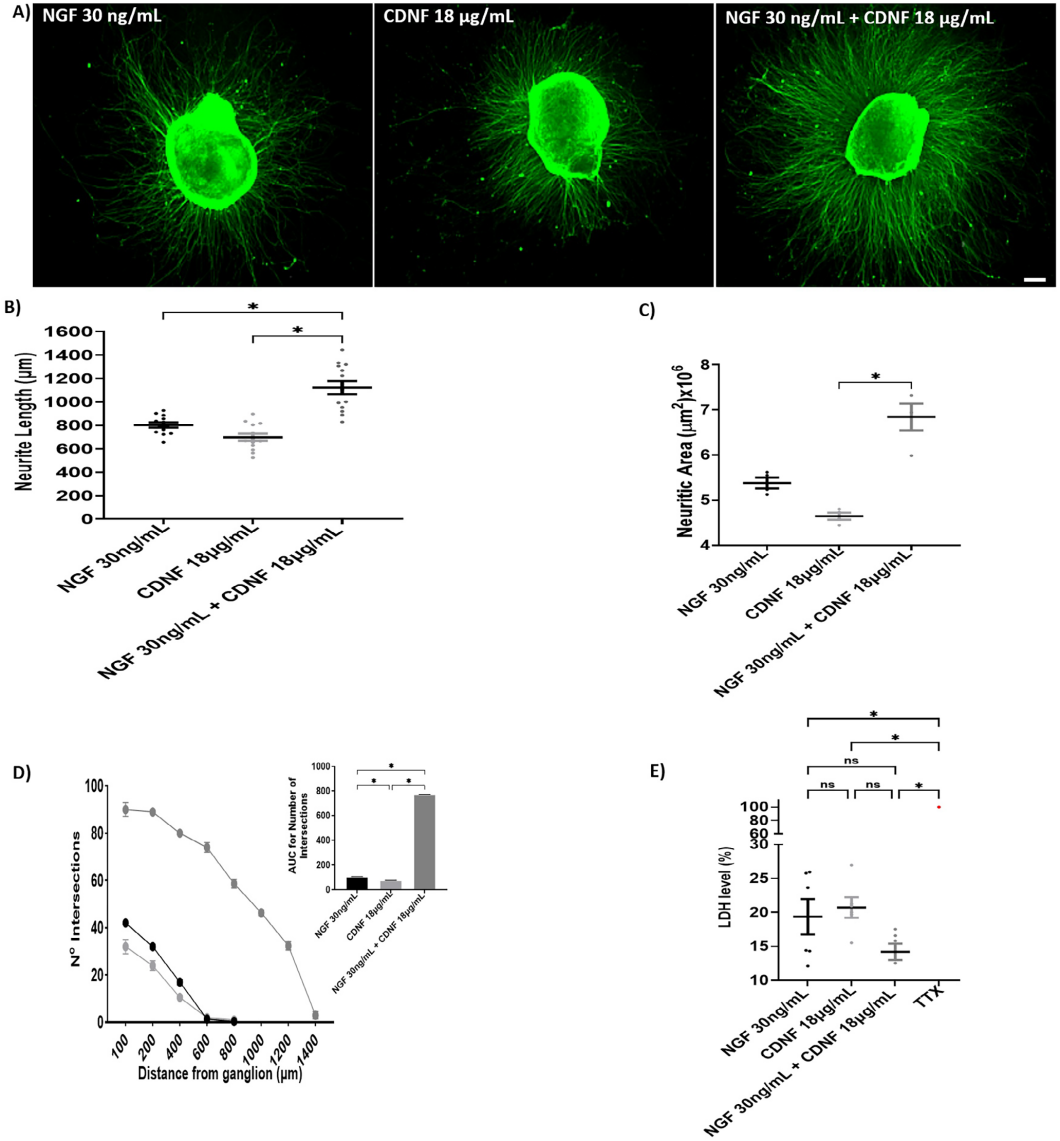
CDNF and NGF work additively to promote neurite outgrowth and enhance cell viability in DRG explants. (A) Representative images of dorsal root ganglia explants cultured in the presence of nerve growth factor (NGF, 30 ng/mL), cerebral dopamine neurotrophic factor (CDNF, 18 μg/mL), or NGF 30 ng/mL + CDNF 18 μg/mL. The cytoskeleton of neurons was labeled with anti‐TuJ‐1 antibody in green. (B–D) Quantification of neurite length, neuritic area, and number of intersections, respectively, from the data presented in panel A. Area under the curve (AUC) relative to number of intersections is shown in the inset. (E) Cell viability measured by LDH assay. Triton X‐100 (TTX) was used as a positive control for complete cell death, with non‐viable cells represented in red. Bar = 750 μm. Data are presented as mean ± SEM from *n* = 3 independent experiments. One‐way ANOVA with Tukey's post hoc test for significance was applied to all analyses, except in graph (C), in which Kruskal–Wallis with Dunn's post hoc test for significance was applied. *p < 0.05, indicates statistical significance.

To investigate this, DRG explants were treated with a combination of NGF and CDNF. As shown in Figure 2, the co‐treatment elicited an additive effect on neuritogenesis compared to the administration of either factor alone. In the presence of both NGF (30 ng/mL) and CDNF (18 μg/mL), neurites extended to an average length of approximately 1100 μm—significantly longer than those observed with NGF (800 μm) or CDNF (700 μm) alone (Figure 2A,B). Similar additive effects were observed in neurite area coverage and network complexity, as indicated by the number of neurite intersections (Figure 2C,D).

Regarding cell viability (Figure 2E), low levels of LDH were found in DRG explants, suggesting that they were already protected in the presence of either CDNF (18 μg/mL) or NGF (30 ng/mL) alone. When these concentrations of NGF and CDNF were combined, cell survival increased very little (LDH level was even lower) without significance.

### 3.3 CDNF Confers Neuroprotection to DRG Explants Under Trophic Deprivation Conditions as NGF

**FIGURE 3:**
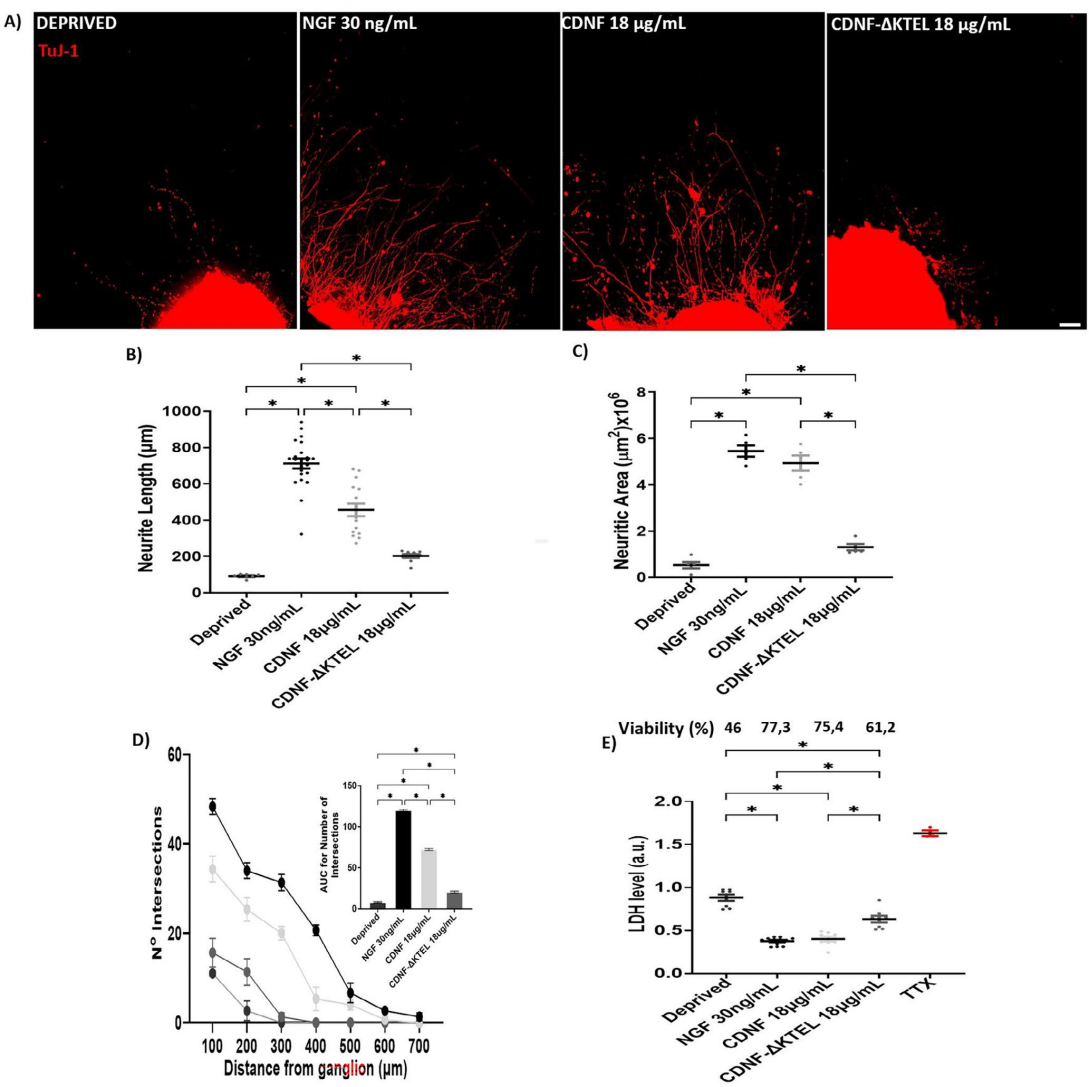
CDNF restores neurite outgrowth in DRG explants after trophic deprivation, whereas CDNF‐ΔKTEL fails to do so. (A) Panel of representative images of dorsal root ganglia (DRG) immunostained with anti‐TuJ‐1 antibody (red). DRG explants were maintained in culture for 24 h with Nerve Growth Factor (NGF, 30 ng/mL), after which the media was changed to a new media without NGF and the explants were kept for 48 h (deprived group), when either NGF (30 ng/mL), cerebral dopamine neurotrophic factor (CDNF, 18 μg/mL), or (C) CDNF‐∆KTEL (18 μg/mL) were added. Neurite length, neuritic area, and number of intersections for the treatments are presented in panels (B–D), respectively. Area under the curve (AUC) relative to Number of Intersections is shown in the inset. (E) Cell viability assays as measured by LDH after deprivation and reconstitution with the same treatments presented in panel (A). Triton X‐100 (TTX) was used as a positive control for complete cell death, with non‐viable cells represented in red. Data are presented as mean ± SEM from *n* = 3 independent experiments. One‐way ANOVA, with Tukey's post hoc test for significance. *p < 0.05 indicates statistical significance.

Neurites undergo significant retraction after 48 h of NGF deprivation, with their average length reduced to 100 μm (Figure 3A,B). Supplementation with NGF (30 ng/mL) effectively restores neurite outgrowth, resulting in an average length of over 700 μm (Figure 3A,B). Similarly, CDNF (18 μg/mL) rescues neurite growth, reaching an average length of 500 μm (Figure 3A,B). In contrast, CDNF‐ΔKTEL (18 μg/mL) fails to restore neurite outgrowth, with an average length of only 200 μm (Figure 3A,B). This trend is consistent with measurements of neurite area (Figure 3C) and network complexity, assessed by the number of intersections (Figure 3D).

The protective effect of CDNF was further confirmed using the LDH release assay. Under trophic deprivation, approximately half of the cells lost viability (46% viable cells). This effect was significantly reversed by the addition of NGF (30 ng/mL) and CDNF (18 μg/mL), which increased cell viability to 77% and 75%, respectively. In contrast, treatment with CDNF‐ΔKTEL conferred lower protection (61% viable cells), a level comparable to the untreated, trophic‐deprived condition (Figure 3E).

### 3.4 CDNF and NGF Activate PI3K Through Distinct Signaling Pathways to Promote Neurite Outgrowth

As demonstrated earlier (Figure 2), NGF and CDNF may act additively to promote neurite outgrowth. To further investigate this interaction, we examined whether these NFs bind to the same receptor and activate overlapping or distinct signaling pathways. Previous studies have shown that CDNF exerts cardioprotective effects through the PI3K/AKT pathway (Maciel et al. 2021), a pathway also implicated in NGF‐induced neuroprotection via its interaction with TrkA (Longo and Massa 2013). Since both NFs appear to depend on PI3K signaling, we also explored whether the mTOR pathway contributes to CDNF's neurotrophic effects, as it does in NGF signaling (Uzdensky et al. 2015; Fahnestock and Shekari 2019; Li et al. 2022).

**FIGURE 4:**
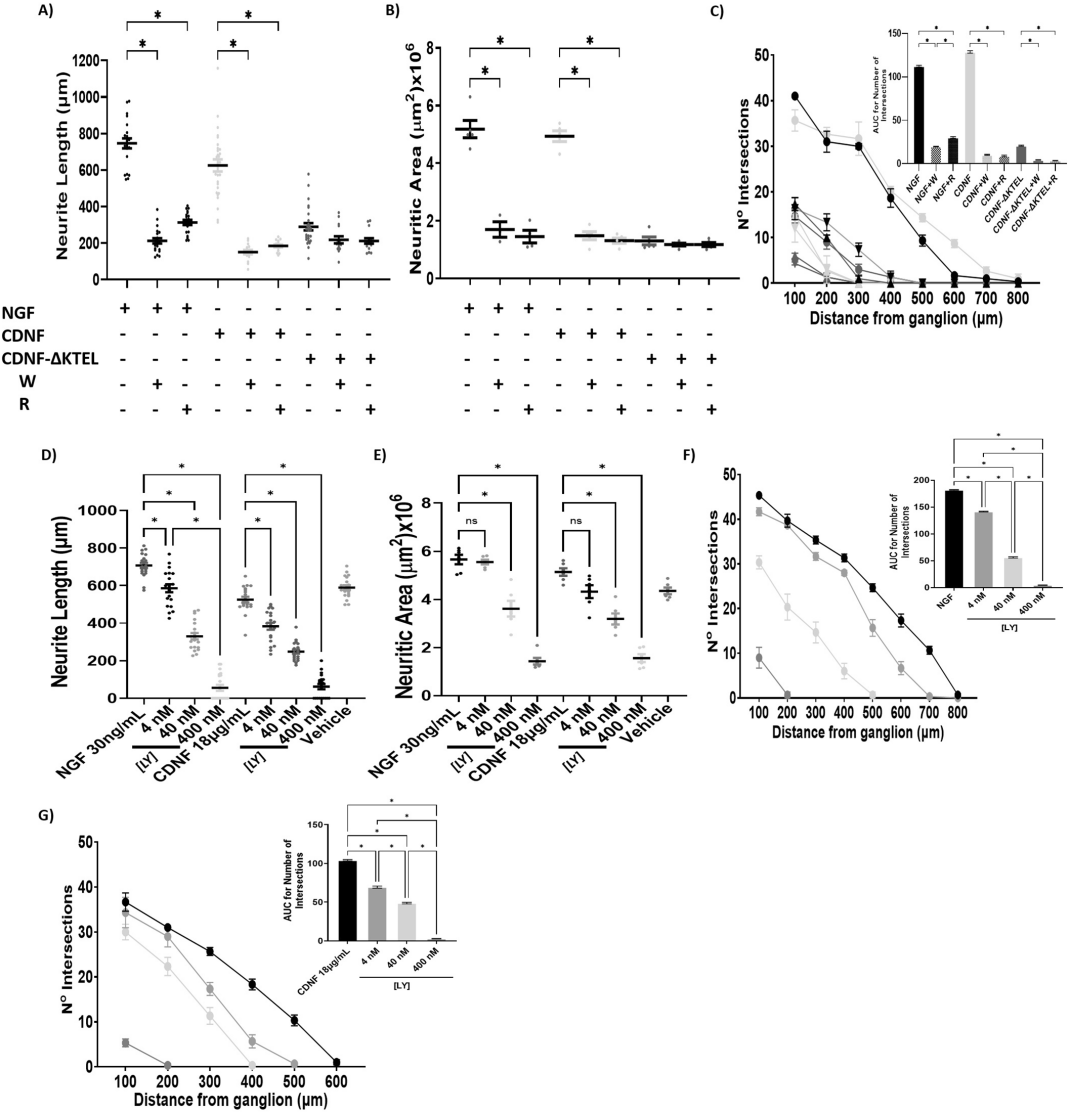
CDNF exerts its trophic activities via the PI3K/AKT/mTOR signaling pathway. Dorsal root ganglia were cultured for 3 days in the presence of nerve growth factor (NGF, 30 ng/mL), cerebral dopamine neurotrophic factor (CDNF, 18 μg/mL), or CDNF‐∆KTEL (18 μg/mL) in the absence or in the presence of Wortmannin (W) (400 nM), LY294002 (LY) (4–400 nM as stated in the panels) or Rapamycin (R) (20 nM), inhibitors of the PI3K/AKT and mTOR, respectively. (A–C) Quantifications of neurite length, neuritic area, and number of intersections, for the data with W and R, as displayed below each panel. (D–G) Quantifications of neurite length, neuritic area, and number of intersections, respectively, for the data with LY294002 in the concentrations mentioned in the panels. Area under the curve (AUC) relative to the number of intersections is shown in the inset. Data are presented as mean ± SEM from *n* = 3 independent experiments. One‐Way ANOVA, with Tukey's post hoc test for significance. *p < 0.05 indicates statistical significance.

As expected, the neuritogenic effect of NGF was almost completely abolished in the presence of either the PI3K inhibitor (Wortmannin; W) or the mTOR inhibitor (Rapamycin; R) (Figure 4A–C, black symbols). Similarly, the neuritogenic effect of CDNF was significantly reduced upon PI3K or mTOR inhibition (Figure 4A–C, light gray symbols), as reflected by decreases in neurite length, neurite area, and network complexity (panels A–C, respectively). Although minimal, the residual activity of CDNF‐ΔKTEL was further diminished in the presence of both inhibitors (Figure 4A–C, dark gray symbols), although with no significance.

To confirm the involvement of the PI3K signaling pathway in the neuritogenic effect promoted by CDNF, we employed an additional PI3K inhibitor, LY294002. As shown in panels D–G, treatment with LY294002 led to a dose‐dependent reduction in neurite outgrowth induced by both NGF (30 ng/mL) and CDNF (18 μg/mL), further supporting the role of the PI3K pathway in mediating the trophic effects of CDNF.

Taken together, these findings indicate that both NGF and CDNF mediate their neuritogenic effects via the canonical PI3K/AKT/mTOR pathway.

To determine whether NGF and CDNF independently activate the PI3K pathway, we examined whether CDNF's neurotrophic activity operates independently of TrkA receptor signaling in DRG neurons. Specifically, we investigated whether CDNF directly activates TrkA or indirectly promotes its activation by inducing NGF secretion.

**FIGURE 5:**
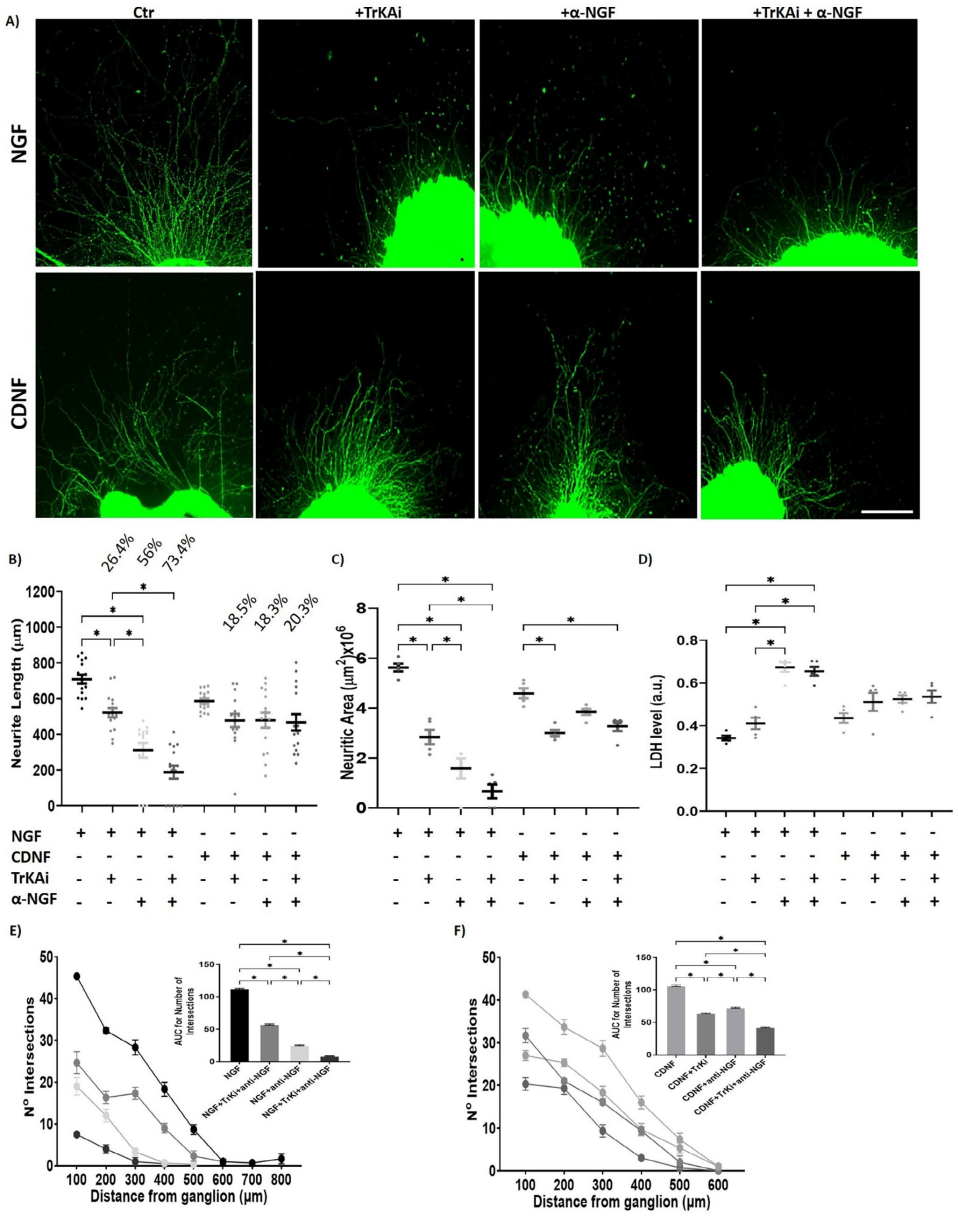
CDNF's trophic activity appears to be independent of both NGF and TrkA receptor signaling. Pharmacological inhibitor (GNF‐5837) of Tropomyosin Receptors kinase (+Trki) or anti‐nerve growth factor (NGF) antibody (+α‐NGF) or both (Trki+α‐NGF) were added to dorsal root ganglia (DRG) explants stimulated with NGF 30 ng/mL or cerebral dopamine neurotrophic factor (CDNF, 18 μg/mL). (A) Representative images of DRG explants immunostained with anti‐TuJ‐1 antibody (green) treated with NGF for 72 h (upper images) or with CDNF (lower images) in the absence (control—Ctr) or in the presence of Trki (+Trki—20 μM), anti‐NGF (+α‐NGF—10 μM) or both (Trki + α‐NGF). (B) Neurite length and (C) neuritic area for the conditions displayed in panel A. (D) Cell toxicity assays measured by LDH for the conditions displayed in panel A. Number of Intersections in the presence of NGF (E) or CDNF (F) for the conditions displayed in panel A. Area under the curve (AUC) relative to number of intersections in shown in the inset. The percentage of neurite outgrowth inhibition for each condition is shown at the top in (B). Scale bar = 300 μm. Data are presented as mean ± SEM from *n* = 3 independent experiments. One‐way ANOVA, with Tukey's post hoc test for significance. *p < 0.05 indicates statistical significance.

As seen in Figure 5, although NGF's neurotrophic activity was significantly reduced in the presence of the pan‐Trk inhibitor (GNF‐5837), anti‐NGF antibody, and when both were applied together (panels A–C and E), CDNF‐treated DRG explants showed only minimal reductions in neuritogenic activity under these conditions (panels A–C, E, and F). A similar trend was observed in CDNF's neuroprotective activity, as assessed by the LDH assay (Figure 5D), further supporting the notion that CDNF functions independently of TrkA signaling.

Since in panel 5B the pan‐Trk inhibitor only decreases the activity of NGF by 26%, a dose–response assay with this pan‐Trk inhibitor (GNF‐5837) was performed (Figure S2). As the concentration of the inhibitor increased, all measured neuritogenic parameters declined significantly under NGF stimulation, with a marked reduction observed at the highest concentration (100 μM), indicating strong dependence on Trk signaling. In contrast, CDNF‐induced neuritogenesis was only mildly affected, even at the highest inhibitor concentration.

These results strongly support the conclusion that CDNF promotes neurite outgrowth through a Trk‐independent mechanism (Figure S2).

### 3.5 The KDEL Receptor Is a Strong Candidate for Mediating CDNF Signaling at the Plasma Membrane of DRG Neurons

Our findings suggest that NGF and CDNF independently activate the PI3K pathway, which is essential for neurite outgrowth and neuroprotection. Furthermore, the lack of neurotrophic activity observed with CDNF‐ΔKTEL indicates that CDNF's effects are mediated via the KDEL‐R.

To confirm the interaction between CDNF and KDEL‐R at the plasma membrane, we assessed whether CDNF colocalizes with KDEL‐R on the membrane surface using ^FITC^CDNF and ^FITC^CDNF‐ΔKTEL.

**FIGURE 6:**
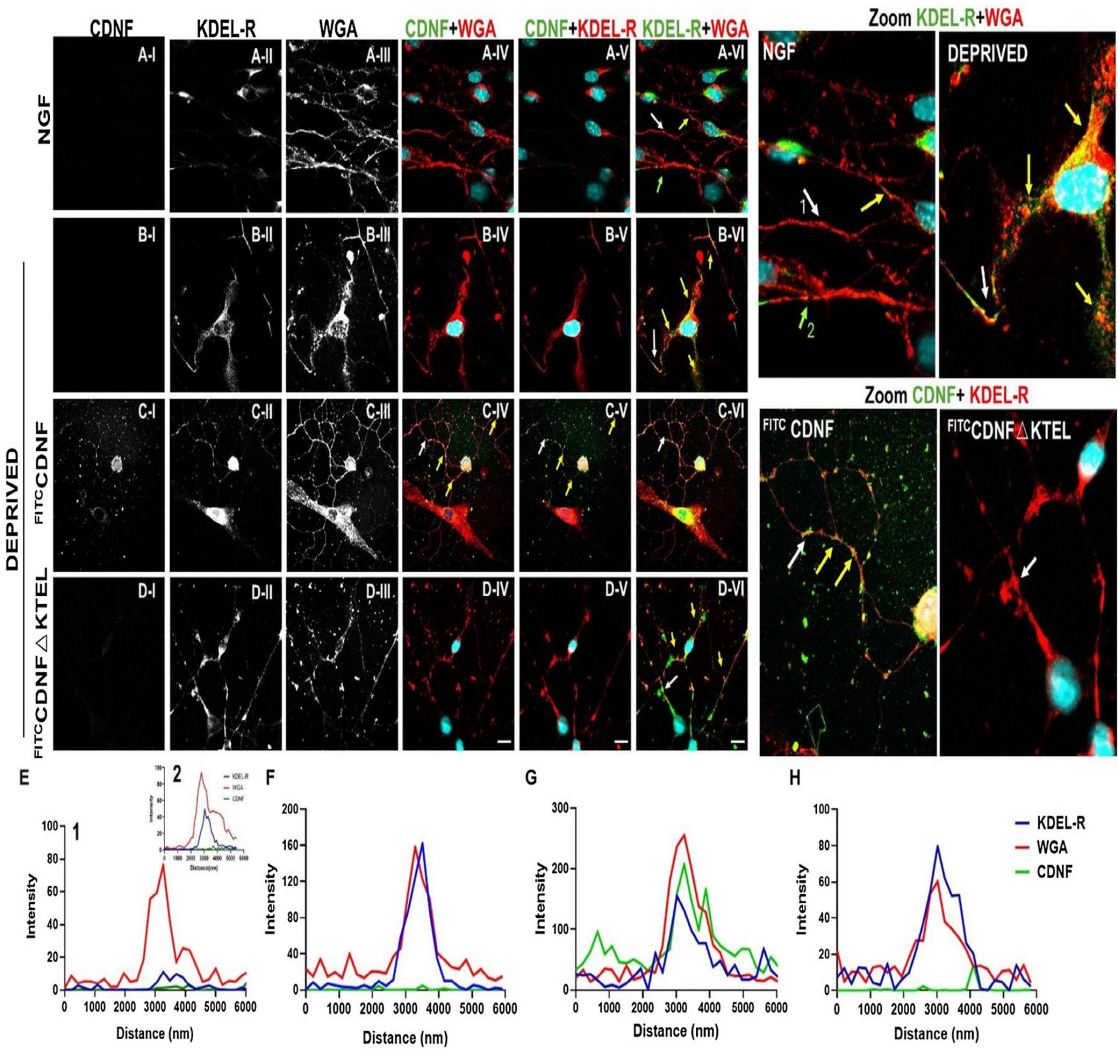
Under NGF deprivation stress, KDEL‐R translocates to the plasma membrane of DRG explants, where it functions as a receptor for exogenous CDNF. Representative confocal immunocytochemistry images of cells treated with nerve growth factor (NGF) (A) or stressed by the suppression NGF for 24 h (B–D). ^FITC^cerebral dopamine neurotrophic factor (^FITC^CDNF, 18 μg/mL) or ^FITC^CDNF‐ΔKTEL (18 μg/mL) was added after this time to see whether they bind to the KDEL‐R displaced to the plasma membrane (panels C and D). Individual channels are shown in shades of gray for better visualization of KDEL‐R (antibody against KDEL‐R1) or ^FITC^CDNF bound to the plasma membrane (wheat germ agglutinin, WGA). The colored images show double colocalization of ^FITC^CDNF or ^FITC^CDNF‐ΔKTEL and WGA; KDEL‐R and ^FITC^CDNF or ^FITC^CDNF‐ΔKTEL; and WGA and KDEL‐R. Cell nuclei were stained with DAPI in blue. The color of each label is indicated in the images. Yellow and green (only in panel A) arrows indicate the regions of colocalization between markers and white or green (only in panel A) arrows indicate the region used to construct the panels E–H (Histograms 1 and 2 in panel E represent the regions pointed by the white and green arrows, respectively, in the NGF zoomed image). (E–H) Histograms showing the localization of each fluorescence signal arising from WGA (red), KDEL‐R (blue), and ^FITC^CDNF (green) or ^FITC^CDNF‐ΔKTEL (green). Scale bar = 10 μm.

Under non‐deprived condition (NGF), KDEL‐R was predominantly localized in the perinuclear region, consistent with its presence in the Golgi/ER, although there is a population already at the plasma membrane (Figure 6AII,V,VI, and zoomed NGF image). Although cells were not permeabilized before antibody treatment, the fixation process may have transiently disrupted the plasma membrane, allowing for limited antibody penetration. Despite being a rare event, panel A‐VI and the corresponding zoomed image display a region of colocalization between WGA fluorescence (red; a membrane marker) and KDEL‐R (green), as indicated by the green arrow, suggesting the presence of KDEL‐R at the plasma membrane. Panel E displays two histograms: Histogram 1 (reflecting the white arrow in the zoomed image) corresponds to regions of the plasma membrane labeled by WGA that do not colocalize with KDEL‐R, whereas Histogram 2 (reflecting the green arrow in the zoomed image) represents regions where colocalization between WGA and KDEL‐R is observed.

However, under stress conditions, such as NGF deprivation (Figure 6B), multiple regions show that KDEL‐R relocates to the plasma membrane, particularly within neurites (deprived group, panels B‐II, B‐VI, and the zoomed image of the deprived condition). This observation is further supported by the colocalization histograms (panel F), where the fluorescence signals of WGA and KDEL‐R overlap within the same plane. This colocalization strongly suggests that KDEL‐R translocates to the plasma membrane in response to stress.

When ^FITC^CDNF was added to stressed cells, shaded images revealed the presence of both KDEL‐R and CDNF at the cell periphery (panels C I–III). In the merged images (panels C IV–VI), multiple regions along the plasma membrane exhibited colocalization of KDEL‐R and ^FITC^CDNF (zoomed ^FITC^CDNF image). This colocalization is further confirmed in panel G, where histograms show overlapping fluorescence signals for the receptor (blue), CDNF (green), and plasma membrane (red), indicating that CDNF binds to KDEL‐R at the plasma membrane under stress conditions.

In contrast, when ^FITC^CDNF‐ΔKTEL was added to stressed cells, shaded images revealed the presence of KDEL‐R at the cell periphery (panel D‐II), but no detectable signal for the NF (panel D‐I). This lack of colocalization is further confirmed in the merged images (panels D IV–VI), the zoomed ^FITC^CDNF‐ΔKTEL image, and the histograms in panel H.

These findings strongly support the notion that KDEL‐R translocates to the plasma membrane in neurons experiencing stress because of neurotrophic deprivation. Furthermore, they reinforce the hypothesis that KDEL‐R acts as the putative membrane receptor for exogenous CDNF, consistent with the observation that CDNF‐ΔKTEL lacks neurotrophic activity.

### 3.6 CDNF Provides Neurotrophic Support to Dissociated DRG Cell Cultures and Exhibits Stronger Protective Effects on Neurons Compared to Schwann Cells (SCs)

**FIGURE 7:**
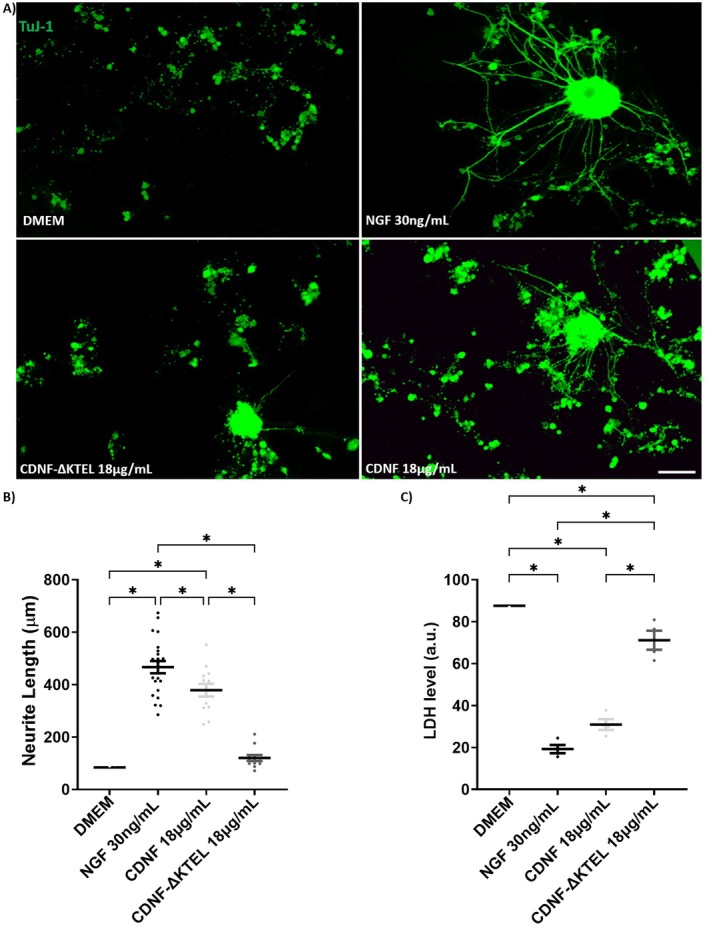
CDNF promotes neuritogenesis in dissociated DRG cell cultures. (A) Epifluorescence images of mouse P1‐dorsal root ganglia dissociated cells placed in culture for 3 days in DMEM/F‐12 plus 10% fetal bovine serum only (DMEM group), or in the presence of nerve growth factor (NGF, 30 ng/mL), CDNF (18 μg/mL), or CDNF‐∆KTEL (18 μg/mL). The cytoskeleton was labeled with anti‐TuJ‐1 antibody (green). (B) Quantification of neurite length. (C) LDH viability assays measured under the same conditions described in panel A. Scale bar = 100 μm. Data are presented as mean ± SEM from *n* = 3 independent experiments. One‐way ANOVA, with Tukey's post hoc test for significance. *p < 0.05 indicates statistical significance.

As shown in Figure 7A, in the absence of exogenous trophic support, dissociated DRG cells exhibited no neurite outgrowth, forming only small, round cell clusters, indicative of neuronal degeneration. This may be due to the predominance of SCs, as the serum‐containing medium favors their development.

However, when NGF (30 ng/mL) or CDNF (18 μg/mL) was added to the dissociated cell cultures (Figure 7A), neurons extended long neurites, mirroring the trends observed in DRG explants (Figure 1A). Under these conditions, neurites reached an average length of approximately 450 μm with NGF and 390 μm with CDNF, whereas CDNF‐ΔKTEL failed to support neurite outgrowth, resulting in lengths of less than 200 μm (Figure 7A,B).

Additionally, cell death was significantly reduced in the presence of NGF and CDNF, but not with CDNF‐ΔKTEL (Figure 7C), further emphasizing the neuroprotective role of CDNF.

Since CDNF promotes neuritogenesis and provides neuroprotection in both DRG explants and dissociated cell cultures, our next objective was to determine whether its beneficial effects are specific to neurons, SCs, or both.

**FIGURE 8:**
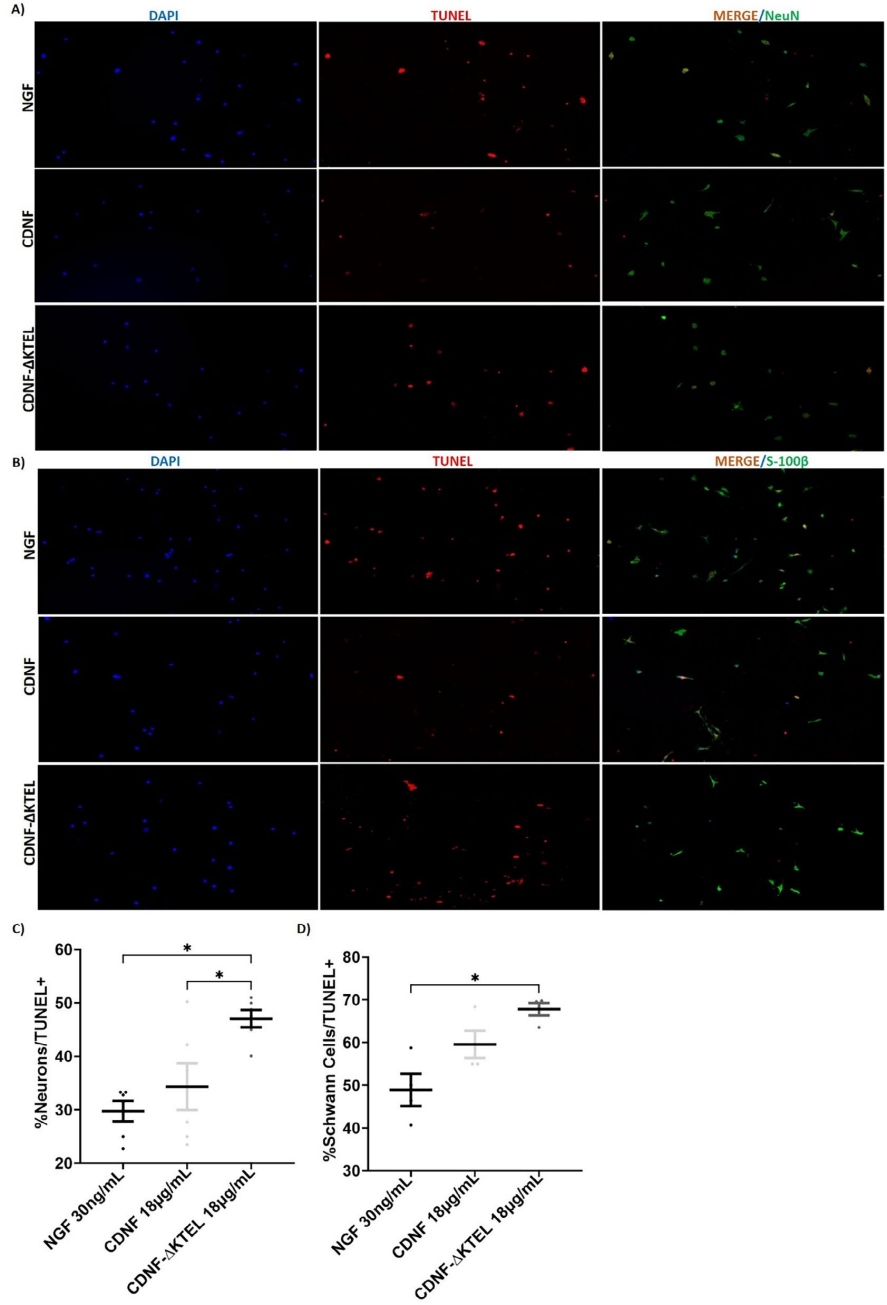
Like NGF, CDNF exhibits stronger cytoprotective effects on neurons in culture compared to Schwann cells (SCs). (A, B) Photomicrograph of dorsal root ganglia dissociated cells stained with DAPI (blue), TUNEL‐positive cells (red), and MERGE (NeuN or S100β, panels A and B, respectively). Cells were treated with nerve growth factor (NGF, 30 ng/mL), cerebral dopamine neurotrophic factor (CDNF, 18 μg/mL), or CDNF‐∆KTEL (18 μg/mL). In (C), quantification of TUNEL‐positive neurons and in (D) TUNEL‐positive Schwann cells. NeuN is a specific marker for neurons and S100β for Schwann cells. Scale bar = 100 μm. Data are presented as mean ± SEM from *n* = 4 independent experiments. One‐way ANOVA, with Tukey's post hoc test for significance. *p < 0.05 indicates statistical significance.

To investigate this, dissociated DRG cells were immunostained with S‐100β and NeuN antibodies, which specifically label SCs and neurons, respectively. Cell viability was then assessed by quantifying the number of TUNEL‐positive (dead) cells under each condition (Figure 8).

The results indicate that both NGF and CDNF provide cytoprotection to neurons and SCs (Figure 8C,D, respectively). However, the protective effect is significantly stronger in neurons, as evidenced by markedly lower cell death rates in the presence of either NF.

## 4 Discussion

CDNF is an unconventional NF that primarily resides in the lumen of the ER. Along with its paralog MANF, CDNF modulates the UPR from within the ER, preventing prolonged activation of pro‐apoptotic pathways and inflammation (Eesmaa et al. 2022). This modulation helps alleviate ER stress, a major contributor to cellular injury and apoptosis.

Notably, ER stress has been implicated in several neurodegenerative diseases, including Parkinson's disease (Yoshida 2007; Colla et al. 2012; Ghemrawi and Khair 2020; Muneeb et al. 2025), positioning CDNF as a promising therapeutic candidate.

At the C‐terminus of CDNF, the presence of a KTEL sequence enables its interaction with the KDEL receptor (KDEL‐R), which recycles proteins from the Golgi back to the ER, preventing their secretion and maintaining intracellular homeostasis (Raykhel et al. 2007).

Under stress conditions, such as calcium imbalance and oxidative stress, CDNF can be secreted from cells, functioning as a conventional NF. In this extracellular role, it exerts paracrine and autocrine effects or acts as a cardiomyokine, providing protective and restorative benefits to the heart (Maciel et al. 2021).

Interestingly, CDNF has been detected in human serum, suggesting that it can be actively secreted (Galli et al. 2019). However, the precise mechanisms and triggers driving CDNF secretion remain poorly understood. Studies have identified factors such as tunicamycin, thapsigargin, and ischemia/reperfusion (I/R) as potential inducers, although their effects appear to be cell type‐dependent (Maciel et al. 2021; Eesmaa et al. 2022). Therefore, investigations into CDNF's therapeutic potential must consider its dual role as both an intracellular modulator and an extracellular signaling molecule. The identification of CDNF's putative extracellular receptor and its downstream signaling pathways remains elusive but is crucial for a comprehensive understanding of the mechanisms underlying this emerging family of NFs.

Notably, a CDNF variant lacking the KTEL sequence (CDNF‐ΔKTEL) is more readily secreted from cells compared to the full‐length protein (Galli et al. 2019). In our previous study on cardiac context, we demonstrated that exogenously administered full‐length CDNF, but not CDNF‐ΔKTEL, protects cardiomyocytes from ER stress and I/R‐induced infarction in both isolated hearts and in vivo models. This cardioprotective effect is mediated through the activation of the PI3K/AKT signaling pathway (Maciel et al. 2021). The lack of activity observed with the truncated CDNF variant suggests that the KDEL‐R at the plasma membrane plays a crucial role in mediating CDNF's protective effects.

In this study, we further explored how CDNF exerts its neuroprotective activity as a canonical NF, using DRG neurons as a model and comparing its effects to NGF, one of the most extensively studied NFs.

Our findings revealed in a pioneering manner that, like NGF, CDNF exhibits both neuroprotective and neurotrophic effects in DRG neurons, in contrast to data found by Lindholm and colleagues, where they did not observe effects in DRG and superior cervical ganglia (Lindholm et al. 2007). In their model, they explored the effects of CDNF in a nanogram range, and they used DRG from E14 and E15 mice. We were able to promote survival and neurite outgrowth in DRG cells only by increasing the concentration of CDNF (Figure 1 and Supplementary Figure S1) with P0–P3 mice. However, these effects are independent of TrkA, the receptor tyrosine kinase associated with NGF, suggesting that CDNF interacts with a distinct receptor. We propose that KDEL‐R is the receptor mediating CDNF's effects in DRG neurons, as it translocates to the plasma membrane of trophically deprived DRG cells, where it colocalizes with added ^FITC^CDNF (Figure 6).

Despite engaging different receptors, both NGF/TrkA and CDNF/KDEL‐R rely on the PI3K/AKT/mTOR signaling pathway, a well‐established survival‐promoting cascade. This suggests a potential link between plasma membrane‐localized KDEL‐R and PI3K activation. However, it remains unclear whether KDEL‐R, whose structure resembles that of a GPCR, directly activates PI3K or does so indirectly through an intermediary signaling molecule.

Previous studies have shown that CDNF, when added to the culture medium of non‐stressed dopaminergic neurons, lacks the survival‐promoting activity observed with GDNF (Eesmaa et al. 2022). In contrast, our findings demonstrate that CDNF provides trophic support to naïve DRG explants, effectively replacing NGF (Figure 1), even under trophic deprivation conditions (Figure 3). To our knowledge, this is the first study to investigate the effects of CDNF on DRG explants, highlighting its previously unrecognized neurotrophic potential in the PNS.

However, previous experiments with postnatal superior cervical ganglion (SCG) explants microinjected with either a CDNF plasmid vector or CDNF protein demonstrated increased resistance to tunicamycin‐induced stress compared to uninjected SCG (Eesmaa et al. 2022). Notably, in this study, SCG explants were cultured in the presence of NGF, raising the question of whether exogenously added CDNF could exert a similar neurotrophic effect on sympathetic neurons of the SCG, as observed in sensory neurons of the DRG. Investigating this could provide further insight into CDNF's broader neuroprotective role in the PNS.

Despite the limited studies on CDNF's effects in DRG neurons, a report has investigated the role of MANF in this context (Mätlik et al. 2015). In this study, MANF overexpression in DRG neurons conferred protection against etoposide‐ and thapsigargin‐induced stress (Mätlik et al. 2015). Interestingly, although the overexpression of MANF‐Δ‐RTDL localized the protein to the ER, the deletion of its last four amino acids abolished its cytoprotective activity. However, in the same study, intracortical injection of MANF or MANF‐Δ‐RTDL prior to cerebral artery occlusion/reperfusion resulted in similar levels of neuroprotection, suggesting that in this context, the KDEL‐binding sequence is not essential for MANF's biological activity.

This finding aligns with more recent observations that the neuroprotective activity of exogenously added MANF is mediated through its interaction with sulfatides in the membrane and the neuroplastin receptor (Bai et al. 2018; Yagi et al. 2020). However, other studies have demonstrated that the KDEL‐R plays a crucial role in retaining MANF at the plasma membrane of neuroblastoma cells and rat cortical neurons following ER calcium depletion (Henderson et al. 2013).

Despite the high structural similarity between MANF and CDNF, only MANF interacts with sulfatides and neuroplastin at the cell membrane (Bai et al. 2018). The interaction with sulfatide depends on lysine residue K112, which is absent in CDNF, where it is replaced by leucine. Notably, a MANF K112L mutant exhibited a marked reduction in sulfatide binding and a weakened survival‐promoting effect (Bai et al. 2018), highlighting the importance of this residue in MANF's neuroprotective mechanism.

As discussed, the identity of the membrane receptor for exogenous CDNF and MANF remains a subject of debate. However, in this study, we provide evidence that under trophic deprivation, KDEL‐R migrates to the plasma membrane of dissociated DRG cells, where it colocalizes with ^FITC^CDNF, but not with ^FITC^CDNF‐ΔKTEL (Figure 6). The presence of KDEL‐R at the plasma membrane has been reported in various cell types—even under non‐stressed conditions—which may help explain the neurotrophic effects of CDNF (Henderson et al. 2013; Lindahl et al. 2014; Becker et al. 2016). Our findings indicate that a fraction of KDEL‐R is constitutively present at the plasma membrane, even in the absence of cellular stress (Figure 6A).

Notably, this truncated version of CDNF lacks neurotrophic activity in DRG cells, further emphasizing the role of the KDEL‐R binding sequence in CDNF function. To our knowledge, this is the first observation of KDEL‐R at the plasma membrane of DRG cells under trophic deprivation, where it directly interacts with CDNF. This finding strongly supports the hypothesis that KDEL‐R serves as the putative receptor for CDNF in neurons, providing new insights into its mechanism of action as an NF.

Although NGF activates PI3K via its high‐affinity receptor tyrosine kinase TrkA (Huang and Reichardt 2003; Zhang et al. 2017), CDNF activates PI3K through its interaction with the KDEL‐R. Currently, it is unclear whether CDNF binding to plasma membrane‐localized KDEL‐R directly triggers PI3K autophosphorylation or if this process involves an intermediary kinase activated upon KDEL‐R engagement. PKA is a potential candidate, as KDEL‐R is a seven‐transmembrane protein with structural similarities to G‐protein‐coupled receptors (GPCRs). Alternatively, KDEL‐R might transactivate an unknown receptor, which in turn could activate PI3K, suggesting a more complex signaling mechanism. Interestingly, KDEL‐R can be phosphorylated by PKA at Serine 209; however, the precise subcellular location where this phosphorylation occurs remains unknown (Cabrera et al. 2003).

In summary, CDNF's neurotrophic activity is distinct and independent of classical neurotrophins like NGF and BDNF, which rely on receptor tyrosine kinase pathways. This unique mechanism of action underscores CDNF's therapeutic potential in the PNS. The distinct properties of CDNF open new therapeutic avenues for neurodegenerative and neuroinflammatory diseases. Its ability to protect neurons under stress, restore function in damaged neurons, and operate independently of classical neurotrophin pathways makes it a promising candidate for conditions where conventional NFs show limited efficacy.

Furthermore, CDNF's dual neurotrophic and neuroprotective roles in both the central and PNSs position it as an ideal candidate for a wide range of clinical applications, including the treatment of Parkinson's disease, peripheral neuropathies, and chronic pain syndromes.

In conclusion, the present study shows for the first time that CDNF promotes trophic actions in the PNS by supporting viability, survival, and stimulating neurite outgrowth of dorsal root ganglion neurons. CDNF represents a novel class of NFs with broad neuroprotective and neurorestorative capabilities. By targeting key pathways involved in ER stress regulation and neuronal survival, CDNF offers significant potential for treating neurological disorders where other neurotrophins fall short. Continued research and clinical development will further elucidate its mechanisms of action and expand its therapeutic applications in neurodegenerative and neuroinflammatory conditions.

## Author Contributions

**Raphael de Siqueira Santos:** conceptualization, methodology, data curation, investigation, validation, formal analysis, writing – original draft, software. **Flávia Natale Borba:** methodology, investigation. **Dahienne Ferreira de Oliveira:** methodology, investigation, formal analysis. **Marcelo Felippe Santiago:** investigation, formal analysis, methodology. **Alexandre Martins do Nascimento:** investigation, writing – review and editing, validation, software, data curation. **Deborah Schechtman:** conceptualization, supervision, funding acquisition, resources, writing – review and editing. **Debora Foguel:** supervision, funding acquisition, visualization, project administration, resources, writing – original draft, conceptualization, methodology.

## Conflicts of Interest

The authors declare no conflicts of interest.

## Peer Review

The peer review history for this article is available at https://www.webofscience.com/api/gateway/wos/peer‐review/10.1111/jnc.70194.

## Supporting information


**Data S1:** Spreadsheet containing details on all statistical tests applied for analysis. Data include type of test, post hoc test, sample space (N), degrees of freedom, statistics summary, mean differences, 95% confidence intervals and exact *p*‐values.

**Figure S1:** CDNF promotes concentration‐dependent effects in DRG neurons. To determine whether cerebral dopamine neurotrophic factor (CDNF) exerts a biological effect on dorsal root ganglia (DRG) neurons, a concentration‐response curve was performed using CDNF concentrations ranging from 0.18 μg/mL to 180 μg/mL. (A) Quantification of neurite length across conditions. (B) LDH levels detected in the culture medium, indicative of cytotoxicity under each treatment condition. Data are presented as mean ± SEM from *n* = 3 independent experiments. Statistical analysis was performed using one‐way ANOVA followed by Tukey's post hoc test. *p < 0.05 indicates statistical significance.
**Figure S2:** The pan‐Trk inhibitor GNF‐5837 robustly suppresses NGF‐induced, but not CDNF‐induced, neurotrophic effects in a dose‐dependent manner. Dorsal root ganglia (DRG) cultures were treated with increasing concentrations of the pan‐Tropomyosin receptors inhibitor GNF‐5837 (Trki; 20, 40, or 100 μM) in the presence of either nerve growth factor (NGF) or cerebral dopamine neurotrophic factor (CDNF). (A) Quantification of neurite length. (B) Measurement of the neuritic area under each condition. Data shown as mean ± SEM of *n* = 4 independent experiments. Statistical analysis was performed using one‐way ANOVA followed by Tukey's post hoc test. *p < 0.05 indicates statistical significance.

## Data Availability

All data needed to evaluate the conclusions are present in the main manuscript or the Supporting Information.
