# Supplementary material for: Cerebral Dopamine Neurotrophic Factor (CDNF) Acts as a Trophic Factor Promoting Neuritogenesis in the Dorsal Root Ganglia (DRG) Neurons Through Activation of the PI3K Signaling Pathway
Source: J Neurochem. 2025 Aug 19;169(8):e70194. doi: 10.1111/jnc.70194 (PMC12362333; doi:10.1111/jnc.70194)
Supplement: Supplementary file 2 — Figure S1: CDNF promotes concentration‐dependent effects in DRG neurons. To determine whether cerebral dopamine neurotrophic factor (CDNF) exerts a biological effect on dorsal root ganglia (DRG) neurons, a concentration‐response curve was performed using CDNF concentrations ranging from 0.18 μg/mL to 180 μg/mL. (A) Quantification of neurite length across conditions. (B) LDH levels detected in the culture medium, indicative of cytotoxicity under each treatment condition. Data are presented as mean ± SEM from n = 3 independent experiments. Statistical analysis was performed using one‐way ANOVA followed by Tukey's post hoc test. *p < 0.05 indicates statistical significance. Figure S2: The pan‐Trk inhibitor GNF‐5837 robustly suppresses NGF‐induced, but not CDNF‐induced, neurotrophic effects in a dose‐dependent manner. Dorsal root ganglia (DRG) cultures were treated with increasing concentrations of the pan‐Tropomyosin receptors inhibitor GNF‐5837 (Trki; 20, 40, or 100 μM) in the presence of either nerve growth factor (NGF) or cerebral dopamine neurotrophic factor (CDNF). (A) Quantification of neurite length. (B) Measurement of the neuritic area under each condition. Data shown as mean ± SEM of n = 4 independent experiments. Statistical analysis was performed using one‐way ANOVA followed by Tukey's post hoc test. *p < 0.05 indicates statistical significance. [file JNC-169-0-s001.pdf]

Cerebral Dopamine Neurotrophic Factor (CDNF) acts as a trophic factor promoting neuritogenesis in the dorsal root ganglia (DRG) neurons through activation of the PI3K signaling pathway

Raphael de Siqueira Santos1*, Flávia Natale Borba1, Dahienne Ferreira de Oliveira1, Marcelo Felippe Santiago2, Alexandre Martins do Nascimento3, Deborah Schechtman3, and Debora Foguel1*

*Corresponding authors: Raphael de Siqueira Santos (raphael.santos@bioqmed.ufrj.br) and Debora Foguel (foguel@bioqmed.ufrj.br)

1- Instituto de Bioquímica Médica Leopoldo De Meis, Universidade Federal do Rio de Janeiro, Brazil

2- Instituto de Biofísica Carlos Chagas Filho, Universidade Federal do Rio de Janeiro, Brazil

3- Departamento de Bioquímica, Instituto de Química, Universidade de São Paulo, Brazil

Raphael de Siqueira Santos: raphael.santos@bioqmed.ufrj.br; 0000-0001-8942-0850

Flávia Natale Borba: flah.natale@gmail.com; 0009-0006-3771-0390

Dahienne Ferreira: dahienne.oliveira@bioqmed.ufrj.br; 0000-0002-8104-9476

Marcelo Santiago: marcelo.santiago@biof.ufrj.br; 0000-0003-2428-9606

Alexandre Martins do Nascimento: nascimento.alexandre@usp.br; 0000-0002-18090732

Deborah Schechtman: deborah@iq.usp.br; 0000-0002-8874-7023

Debora Foguel: foguel@bioqmed.ufrj.br; 0000-0001-7312-7115

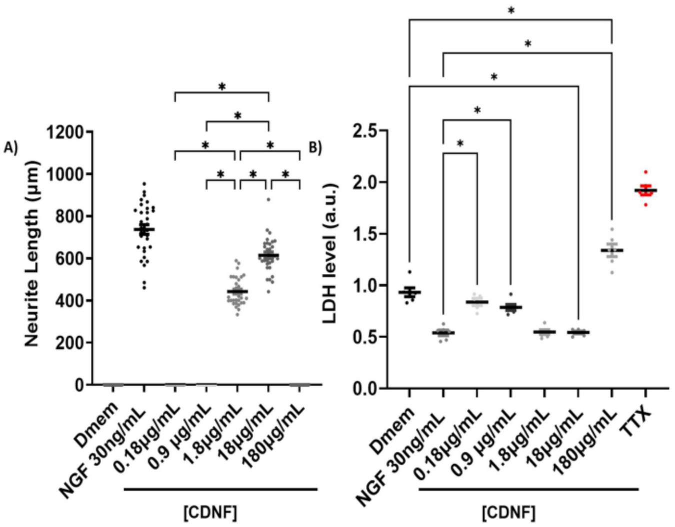

Supplementary Figure 1. CDNF promotes concentration-dependent effects in DRG neurons. To determine whether Cerebral Dopamine Neurotrophic Factor (CDNF) exerts a biological effect on dorsal root ganglia (DRG) neurons, a concentration-response curve was performed using CDNF concentrations ranging from 0.18 µg/mL to 180 µg/mL. (A) Quantification of neurite length across conditions. (B) LDH levels detected in the culture medium, indicative of cytotoxicity under each treatment condition. Data are presented as mean ± SEM from n = 3 independent experiments. Statistical analysis was performed using one-way ANOVA followed by Tukey's post hoc test. *$p < 0.05$ indicates statistical significance.

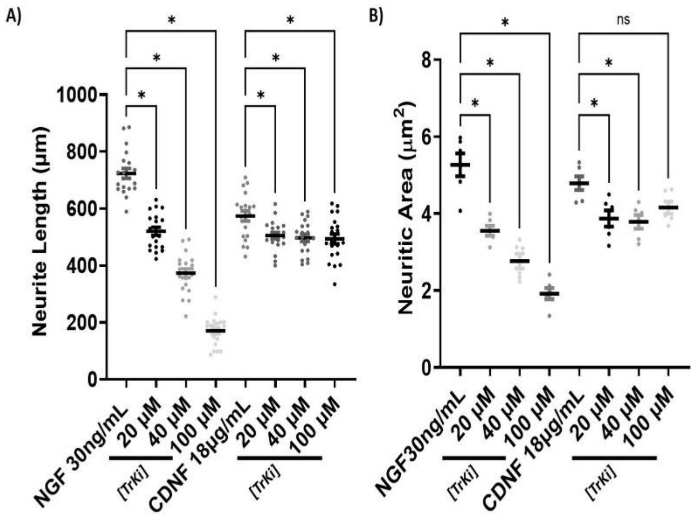

Supplementary Figure 2. The pan-Trk inhibitor GNF-5837 robustly suppresses NGF-induced, but not CDNF-induced, neurotrophic effects in a dose-dependent manner. Dorsal root ganglia (DRG) cultures were treated with increasing concentrations of the pan-Tropomyosin receptors inhibitor GNF-5837 (Trki; 20, 40, or 100 µM) in the presence of either Nerve Growth Factor (NGF) or Cerebral Dopamine Neurotrophic Factor (CDNF). (A) Quantification of neurite length. (B) Measurement of neuritic area under each condition. Data shown as mean ± SEM of n=4 independent experiments. Statistical analysis was performed using one-way ANOVA followed by Tukey's post hoc test. *$p < 0.05$ indicates statistical significance.
